# Molecular characterization of *Escherichia coli* isolated from milk samples with regard to virulence factors and antibiotic resistance

**DOI:** 10.14202/vetworld.2021.2410-2418

**Published:** 2021-09-17

**Authors:** Waleed Younis, Sabry Hassan, Hams M.A. Mohamed

**Affiliations:** 1 Department of Microbiology, Faculty of Veterinary Medicine, South Valley University, Qena, 83523, Egypt; 2 Department of Biology, College of Science, Taif University, P.O. Box 11099, Taif 21944, Saudi Arabia.

**Keywords:** 16S rRNA, antibiotic, *Escherichia coli*, raw milk, serology, virulence

## Abstract

**Background and Aim:**

Raw milk is considered an essential source of nutrition during all stages of human life because it offers a valuable supply of protein and minerals. Importantly, milk is considered a good media for the growth and contamination of many pathogenic bacteria, especially food-borne pathogens such as *Escherichia coli*. Thus, the objective of this study was to characterize *E. coli* and detect its virulence factors and antibiotic resistance from raw milk samples.

**Materials and Methods:**

Raw milk samples (n=100) were collected from different localities in Qena, Egypt, and investigated for the presence of *E. coli* using different biochemical tests, IMViC tests, serotyping to detect somatic antigen type, and molecularly by polymerase chain reaction (PCR) tests. The presence of different virulence and antimicrobial genes (*hly*, *eae*, *stx1*, *stx2, blaTEM*, *tetA(A)*, and *tetB* genes) in *E. coli* isolates was evaluated using PCR.

**Results:**

The results demonstrated that 10 out of 100 milk samples were contaminated with *E. coli*. Depending on serology, the isolates were classified as O114 (one isolate), O27 (two isolates), O111 (one isolate), O125 (two isolates), and untypeable (five isolates) *E. coli*. The sequencing of partially amplified 16S rRNA of the untypeable isolates resulted in one isolate, which was initially misidentified as untypeable *E. coli* but later proved as *Enterobacter hormaechei*. Moreover, antibacterial susceptibility analysis revealed that nearly all isolates were resistant to more than 3 families of antibiotics, particularly to b-lactams, clindamycin, and rifampin. PCR results demonstrated that all *E. coli* isolates showed an accurate amplicon for the *blaTEM* and *tetA(A)* genes, four isolates harbored *eae* gene, other four harbored *tetB* gene, and only one isolate exhibited a positive *stx2* gene.

**Conclusion:**

Our study explored vital methods for identifying *E. coli* as a harmful pathogen of raw milk using 16S rRNA sequencing, phylogenetic analysis, and detection of virulence factors and antibiotic-resistant genes.

## Introduction

*Escherichia coli* is a facultative anaerobe and one of the normal inhabitants in the human and animal intestinal tracts [1]. However, the pathogenic strains of *E. coli* cause many diseases [2]. Recently, pathogenic *E. coli* strains have been categorized using different antibodies for perceiving surface antigens correlated to “183 O-groups (lipopolysaccharide) and 53 H-types (flagellar antigen)” [3]. *E. coli* is differentiated into several pathotypes such as enterotoxigenic *E. coli* (ETEC) (O27), enterohemorrhagic *E.coli* (EHEC) (O111), and enteropathogenic *E.coli* (EPEC)” [4]. In general, these pathotypes encode genes for specific virulence factors that are associated with the attachment and secretion of hemolysins and enterotoxins; however, there is a significant polymorphism presenting the nucleotide sequences of these genes [5].

Dissimilar strains of pathogenic *E. coli* produce several effective toxins, such as Shiga-like toxins. These Shiga toxin-producing *E. coli* (STEC) contribute toward gastroenteritis, bloody diarrhea, and uremic syndrome in infected humans [6]. Other virulence factors elevate the pathogenicity of *E. coli* combined with plasmid-encoded enterohemolysin (*hlyA*), which is frequently associated with acute sickness in individuals [7], and also with intimin (*eaeA*), which is responsible for attachment, adherence, and clustering epithelial cell surfaces [8]. Polymerase chain reaction (PCR) analysis has detected that all strains of *E. coli* possess these virulence genes. However, the procurement of one or more virulence genes does not place bacteria in a harmful grade if that strain has not harbored the proper virulence gene that initiates disease in specific species [9]. “The dispersal of antibacterial resistance genes has been noticed between *E. coli* isolates from human, animal, and environmental sources.” Antimicrobial resistance has been observed as a severe problem worldwide [10]. However, the incidence rates for antibiotic-resistant *E. coli* strains diverge in different environments [10]. Many authors have suggested that the extensive administration of penicillin, tetracyclines, and other sulfa drugs has contributed to the spread of antimicrobial-resistant *E. coli*, specifically those from animal sources [11].

Therefore, the main objectives of this study are to apply a broader array of virulence and antimicrobial-resistant genes that are known to arise in different pathotypes of *E*. *coli* while causing several diseases and to optimize uniplex PCR assays for their detection in milk samples.

## Material and Methods

### Ethical approval

The study was approved by the Animal Ethics Committee for Veterinary Research, Faculty of Veterinary Medicine, South Valley University, Qena, Egypt.

### Study period, location, and detection of *E. coli* in raw milk samples

The study was conducted from February to August 2019. A total of 100 milk samples were collected from different sources (47 samples from dairy farms, 27 samples from retail markets, and 26 samples from farmers’ houses) in Qena, Egypt. These samples were collected under aseptic conditions in a clean, sterile 15 mL falcon tube and transferred instantly in an icebox to the bacteriological laboratory in the Department of Microbiology, Faculty of Veterinary Medicine, South Valley University. One milliliter of milk samples were fed in 9 mL buffer peptone water (Oxoid) and incubated at 37°C for 18-24 h. Subsequently, a sterile loop was used to transfer bacteria from the inoculated buffer peptone water and was inoculated on a MacConkey agar plate (Oxoid). Plates were incubated at 37°C for 24 h. The suspected colonies were inoculated in eosin methylene blue (Oxoid). After the procedure, green metallic sheen colonies were selected for biochemical identification using the IMViC reaction and triple sugar iron test [12].

### Serology of *E. coli* isolates

*E. coli* isolates were serogrouped according to a previous study [13]. The serotyping of *E. coli* isolates was performed by commercially available kits (*E. coli* antisera set 1 for O-antigen, Denka Seiken, Japan), which combined 8 polyvalent sera and 43 monovalent sera.

### Antibiotic susceptibility evaluation of bacterial strains

Antimicrobial susceptibility tests were conducted using the disk diffusion method following the guidelines of a previous research [14]. A ruler premeditated the diameter of the inhibition zone. The sensitivity of each isolate was determined against 12 different antibiotics: Oxacillin (1 μg), trimethoprim (5 μg), tetracycline (30 μg), sulfamethoxazole-trimethoprim (1.25-23.75 μg), gentamicin (10 μg), erythromycin (15 μg), chloramphenicol (30 μg), penicillin G (10 μg), nalidixic acid (30 μg), nitrofurantoin (30 μg), clindamycin (10 μg), and rifampin (30 μg) (Oxoid).

### DNA extraction

DNA extraction was performed using the QIAamp DNA Mini kit (Qiagen GmbH, Germany) with changes to the manufacturer’s recommendations. Briefly, 10 μL of proteinase K and 200 μL of lysis buffer were added to 200 μL of the bacterial suspension, and this mixture was then incubated at 56°C for 10 min. After incubation, the lysate was mixed with 200 μL of 100% ethanol. The mixture was then rinsed and centrifuged after the manufacturer’s recommendations. Nucleic acid was washed with 100 μL of elution buffer accompanied within the kit.

### Detection of 16S *rRNA* gene

The extracted bacterial DNA and controls were amplified with 12.5 μL of Emerald Amp Max PCR Master Mix (Takara, Japan), 1 μL of each primer (20 pmol concentration), 6 μL of DNA template, and 4.5 μL of water to reach a final volume of 25 μL. DNA was amplified (Table-1) in a thermal cycler (Applied Biosystem 2720, California, USA). PCR products were isolated on 1.5% of agarose gel, and DNA amplicons were visualized with ethidium bromide.

**Table 1:** Oligonucleotide primers and polymerase chain reaction conditions.

Target gene	Primers sequences	Amplified segment (bp)	Primary denaturation	Amplification (35 cycles)			Final extension	Reference

				Secondary denaturation	Annealing	Extension		
*16S rRNA*	CCCCCTGGACGAAGACTGAC	401	95°C 8 min	95°C 30 s	58°C 30 s	72°C 30 s	72°C 7 min	[15]
*hly*	ACCGCTGGCAACAAAGGATA AACAAGGATAAGCACTGTTCTGGCT	1177	94°C 5 min	94°C 30 s	60°C 50 s	72°C 1 min	72°C 10 min	[16]
*eaeA*	ACCATATAAGCGGTCATTCCCGTCA ATGCTTAGTGCTGGTTTAGG	248	94°C 5 min	94°C 30 s	51°C 30 s	72°C 30 s	72°C 7 min	[17]
*stx1*	GCCTTCATCATTTCGCTTTC ACACTGGATGATCTCAGTGG	614	94°C 5 min	94°C 30 s	58°C 40 s	72°C 45 s	72°C 10 min	[18]
*stx2*	CTGAATCCCCCTCCATTATG CCATGACAACGGACAGCAGTT	779						
*tetA(A)*	CCTGTCAACTGAGCAGCACTTTG GGTTCACTCGAACGACGTCA	576	94°C 5 min	94°C 30 s	50°C 40 s	72°C 45 s	72°C 10 min	[19]
*blaTEM*	CTGTCCGACAAGTTGCATGA ATCAGCAATAAACCAGC	516	94°C 5 min	94°C 30 s	54°C 40 s	72°C 45 s	72°C 10 min	[20]

### The sequence of partial amplified 16S rRNA gene of untypeable *E. coli* strains

Purified PCR products of partially amplified *16S rRNA* of untypeable *E. coli* strains were sequenced in the forward and reverse directions, as well as in separate reactions, using primers of 16S rRNA, according to the manufacturer’s protocol using the following kits: Big Dye Terminator V.3.1 Cycle sequencing kit (Applied Biosystems), the sequencing cycle purification kit, DyeEx 2.0 Spin kit (Qiagen), and Hi-Di-ionized formamide (Applied Biosystems). DNA sequences were obtained from the Applied Biosystems 3130 (Tokyo, Japan). All the obtained *16S rRNA* gene sequences were submitted to the GenBank. The multiple alignment algorithms in MegAlign (Dnastar, Window version 3.12e) were used to perform the sequence alignment of isolates.

### Phylogenetic analysis

A phylogenetic tree, which relied on the *16S rRNA* gene nucleotide sequence, was a tribute for the untypeable isolates to demonstrate these isolates’ identities and reference strains logged in GenBank using MegAlign from the Lasergene package version 7 (Dnastar).

### Virulence genes in *E. coli* strains

#### Uniplex PCR

Primers for the following genes *hly*, eaeA, blaTEM, *tetA(A)*, and *tetB* were used in a 25 μL reaction containing 12.5 μL of Emerald Amp Max PCR Master Mix (Japan), 1 μL of each primer, 4.5 μL of water, and 6 μL of DNA template. The reaction was conducted in an Applied Biosystem 2720 thermal cycler (Table-1).

### Duplex PCR

Primers for *stx1* and *stx2* were used in a 50 μL reaction containing 25 μL of EmeraldAmp Max PCR Master Mix (Japan), 1 μL of each primer (concentration of 20 pmol), 13 μL of water, and 8 μL of DNA template. The reaction was conducted in a thermal cycler (Applied Biosystem 2720) [15-20] (Table-1).

### Investigation of the PCR products

The uniplex and duplex PCR products were separated by electrophoresis on 1.5% of agarose gel (AppliChem GmbH, Germany) in 1× TBE buffer at room temperature (30ºC) using a gradient of 5 V/cm. For gel analysis, 20 μL of each product was loaded in each gel well. A 100 bp DNA ladder (Qiagen) was used to find out the amplicon sizes. The gel was visualized by a gel documentation system (Alpha Innotech, Biometra, Germany).

## Results

We isolated from 10 out of 100 raw milk samples (10%). Four samples were brought from markets, three samples were brought from dairy farms, and three samples were obtained from farmers’ houses. The serology results of the 10 *E. coli* isolates from the raw milk samples, using antisera against the O-antigen, exhibited that one isolate was O111, one isolate was O27, one isolate was O114, two isolates were O125, and the other five isolates were untypeable (Table-2).

**Table 2:** Antibiotic susceptibility analysis of Escherichia coli serogroups isolated from raw milk.

Samples	Source of milk sample	Serogrouping	Antimicrobial sensitivity											

			O	SXT	TMP	C	TE	F	CN	E	P	NA	DA	RA
1	Dairy farm	O111	R	S	I	I	I	S	I	R	R	I	R	R
2	Retail market	O27	R	S	S	R	R	S	I	R	R	I	R	R
3	Retail market	Untypeable	R	R	R	S	I	R	S	R	R	I	R	R
4	Dairy farm	Untypeable	R	R	I	R	I	R	R	R	R	R	R	R
5	Dairy farm	Untypeable	R	S	S	S	R	S	R	R	R	R	R	R
6	Farmer’s house	Untypeable	R	R	R	R	R	R	R	R	R	I	R	R
7	Farmer’s house	O114	R	R	R	R	R	I	I	R	R	I	R	R
8	Retail market	Untypeable	R	R	R	S	I	R	S	R	R	I	R	R
9	Retail market	O125	R	I	R	R	R	R	R	R	R	R	R	R
10	Farmer’s house	O125	R	S	S	R	R	R	R	R	R	I	R	R

S=Sensitive, R=Resistant, I=Intermediate, O=Oxacillin, SXT=Sulfamethoxazole-trimethoprim, TMP=Trimethoprim, C=Chloramphenicol, TE=Tetracycline, F=Nitrofurantoin, CN=Gentamicin, E=Erythromycin, P=Penicillin, NA=Nalidixic acid, RA=Clindamycin, DA=Rifampin

The antibiotic susceptibility profile of the 10 *E. coli* isolates was determined against 12 different antibiotics according to Clinical Laboratory Standards Institute [14]. All 10 *E. coli* isolates did not show any sensitivity for oxacillin, penicillin, rifampin, and clindamycin. A total of nine isolates were resistant to erythromycin, and six isolates were resistant to trimethoprim. Moreover, five isolates were resistant to trimethoprim-sulfamethoxazole, chloramphenicol, nitrofurantoin, and gentamicin; furthermore, seven isolates did not have any zone of inhibition against tetracycline and four isolates were resistant to nalidixic acid. Four isolates were sensitive to both trimethoprim and trimethoprim-sulfamethoxazole. Three isolates were sensitive to chloramphenicol and nitrofurantoin.

Two isolates were resistant to tetracycline and gentamicin, whereas only one isolate was sensitive to erythromycin. Six isolates cleared intermediary susceptibility toward nalidixic acid, three isolates exhibited intermediate susceptibility to gentamicin, two isolates showed intermediary susceptibility toward chloramphenicol and nitrofurantoin, and only one isolate exhibited intermediary susceptibility toward trimethoprim-sulfamethoxazole and tetracycline (Table-2).

PCR results confirmed the presence of *E. coli* DNA in 10 isolates using housekeeping gene primers (16S rRNA). The presence of different virulence genes (*hly*, *eaeA*, *stx1*, and *stx2*) and antibiotic-resistant genes (*blaTEM*, *tetA(A)*, and *tetB*) was then evaluated in the 10 isolates (Table-3).

**Table 3:** Molecular detection *16S rRNA*, virulence genes, and antimicrobial resistance gene for *Escherichia coli* isolates obtained from raw milk samples.

Sample	Molecular detection							

	*16S rRNA*	*eaeA*	*hly*	*stx1*	*stx2*	*tetA (A)*	*tetB*	*blaTEM*
1	+ †	− ‡	−	−	−	+	−	+
2	+	−	−	−	−	+	−	+
3	+	−	−	−	−	+	−	+
4	+	+	−	−	+	+	+	+
5	+	−	−	−	+	+	+
6	+	+	−	−	−	+	+	+
7	+	−	−	−	−	+	−	+
8	+	−	−	−	−	+	+	+
9	+	+	+	−	−	+	−	+
10	+	+	−	−	−	+	−	+

† mean positive. ‡ mean negative

**Figure-1:**
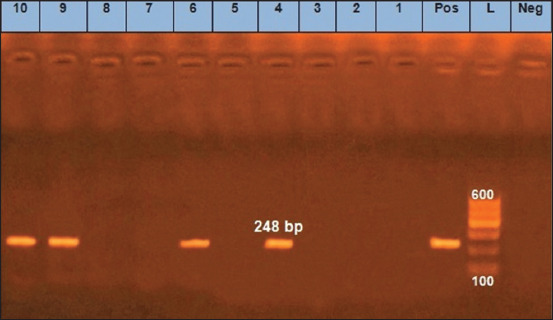
Gel image showing amplification of 248 bp product corresponding to the *eaeA* gene of *E. coli* serogroups isolated from raw milk. Lane (L): 100 bp DNA ladder; lane (Pos): positive control; lane (Neg): Negative control; lanes corresponding to milk samples 1-3, 5, 7, and 8 were negative while samples in lanes 4, 6, 9, and 10 were positive.

**Figure-2:**
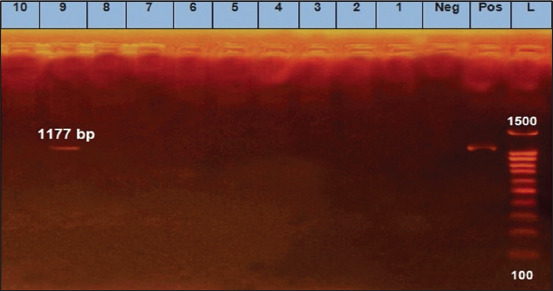
Gel image showing amplification of the 1177 bp band corresponding to the *hyl* gene of *Escherichia coli* serogroups isolated from raw milk samples. Lane (L): 100 bp DNA ladder; lane (Pos): Positive control; lane (Neg): Negative control; lanes corresponding to samples 1-8 and 10 were negative while lane 9 was positive.

**Figure-3:**
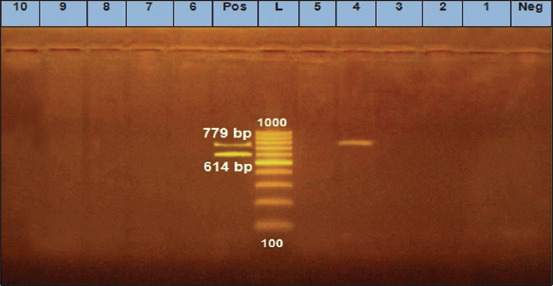
Gel image showing amplification of 614 and 779 bp products corresponding to the *stx1* and *stx2* genes from *E. coli* isolated from raw milk samples. Lane (L): 100 bp DNA ladder; lane (Pos): Positive control; lane (Neg): Negative control; lanes corresponding to samples 1-10 were negative for the *stx1* gene. The milk samples in lane 4 were positive for the *stx2* gene while samples in lanes 1-3 and 5-9 were negative for the *stx2* gene.

**Figure-4:**
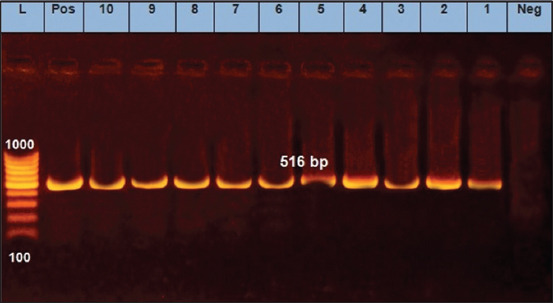
Polymerase chain reaction analysis for detection of the *blaTEM* gene (516 bp product) from *E. coli* isolated from raw milk samples. Lane (L): 100 bp DNA ladder; lane (Pos): Positive control; lane (Neg): Negative control; lanes (1-10) demonstrate that all 10 *E. coli* isolates were positive for the *blaTEM* gene.

**Figure-5:**
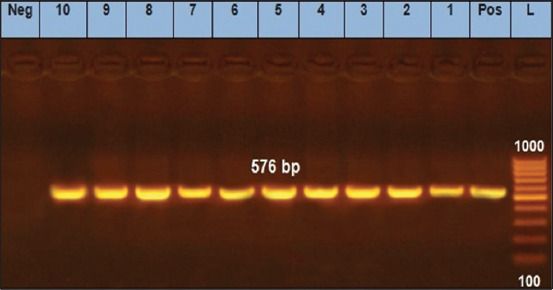
Polymerase chain reaction analysis for detection of the *tetA(A*) gene, amplification of 576 bp product, from *E. coli* isolated from raw milk samples. Lane (L): 100 bp DNA ladder; lane (Pos): Positive control; lane (Neg): Negative control; lanes (1-10) demonstrate that all 10 *E. coli* isolates were positive for the *tetA(A)* gene.

**Figure-6:**
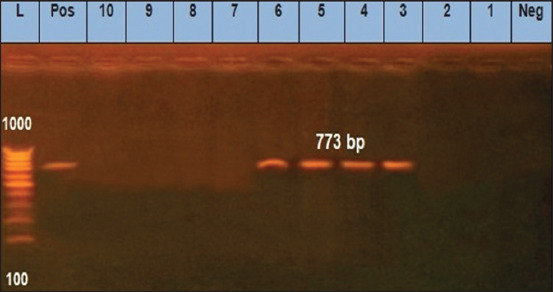
Polymerase chain reaction analysis for detection of the *tetB* gene, amplification of the 773 bp product, from 10 *E. coli* isolated from raw milk samples. Lane (L): 100 bp DNA ladder; lane (Pos): Positive control; lane (Neg): Negative control; samples in lanes (3-6) were positive while samples in lanes 1, 2, and 7-10 were negative.

We found that two *E. coli* isolates (O125) and two untypeable *E. coli* isolates were positive for the *eaeA* gene (Figure-1). Only one *E. coli* isolate (O125) was positive for the *hly* gene (Figure-2). All 10 *E. coli* isolates were negative for the presence of *stx1* and *stx2* genes, except for one untypeable *E. coli* isolate that was positive for *stx2* (Figure-3). As it pertains to the presence of specific antibiotic-resistant genes, we found that all 10 isolates of *E. coli* were positive for the *blaTEM* (Figure-4) and tetA (A) (Figure-5). In addition, four untypeable *E. coli* isolates were positive for the *tetB* (Figure-6).

**Figure-7:**
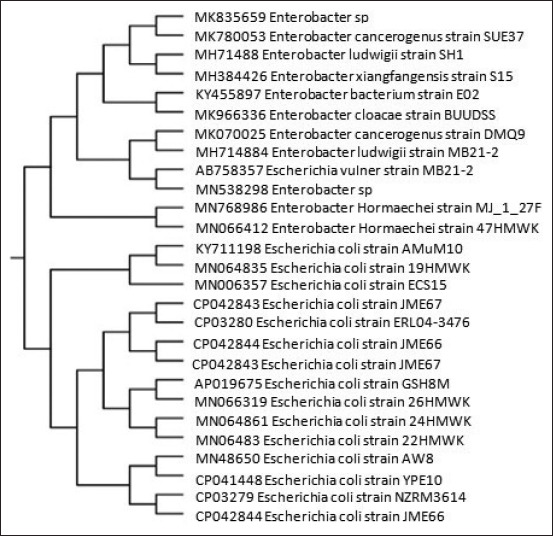
Neighbor-joining tree showing the *16S rRNA* gene phylogenetic relationships of the strains isolated from the raw milk samples and phylogenetically related reference strains from GenBank.

**Figure-8:**
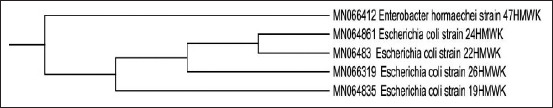
Neighbor-joining tree showing the *16S rRNA* gene phylogenetic relationships of the strains isolated from raw milk samples.

The partially amplified *16S rRNA* gene sequencing results confirmed that four untypeable isolates were of *E. coli* (Table-4). Interestingly, one isolate (number 5) originally classified as *E. coli* was determined to be a strain of *Enterobacter hormaechei* (Table-4) after subjecting this isolate’s sequence to an NCBI Blast search. The neighbor-joining algorithm was used to generate a phylogenetic analysis using MegAlign from the Lasergene package version 7 (Dnastar). The phylogenetic trees, reconstructed from partially amplified *16S rRNA* gene sequences from the five untypeable *E. coli* isolates, were mainly the same altogether. Nevertheless, a slight variety was noticed in branching, as shown in Figure-7. The phylogenetic tree identified groups corresponding to species belonging to the same genus. Our isolates exhibited a high degree of similarity with one other (Figure-8), with similarity percentages ranging between 96.5% and 100%. These isolates were registered on GenBank by accession number (Table-4).

**Table 4:** Accession numbers of the untypeable Escherichia coli isolates.

Isolate No.	Serological identification	Source	*16S rRNA* sequencing	Accession number
1	Untypeable *E. coli*	Retail market	*Escherichia coli*	MN064835
2	Untypeable *E. coli*	Dairy farm	*Escherichia coli*	MN064861
3	Untypeable *E. coli*	Dairy farm	*Escherichia coli*	MN06483
4	Untypeable *E. coli*	Farmer’s house	*Escherichia coli*	MN066319
5	Untypeable *E. coli*	Retail market	Enterobacter hormaechei	MN066412

Furthermore, the degree of similarity and deviation was determined between our isolates and reference strains. Untypeable *E. coli* isolate 1 grouped with *E. coli* MN006357 and *E. coli* KY711198, with an identity percentage of 98.1%. In addition, untypeable *E. coli* isolates 2, 3, and 4 grouped with *E. coli* (AP019675) with similarity percentages of 98.9%, 98.1%, and 98.3%, respectively. The last isolate identified phenotypically as *E. coli* (isolate 5) exhibited the most similarity (98.4% identical) with *E. hormaechei* (MF768986) (Table-4).

## Discussion

The phenotypic identification of raw milk samples revealed a concern with maintaining proper hygienic conditions at the sampling sites. Among the 100 milk samples collected, 10 (10%) were contaminated with *E. coli*. These results are nearly similar to those of Kumar and Prasad [21], who found that 8.14% of the milk samples were contaminated with *E. coli*. da Silva *et al*. [22] isolated *E. coli* from 22.1% of milk samples in Brazil. Disassa *et al*. [23] isolated *E. coli* from 33.9% of cow milk samples in Ethiopia. These studies share the same concern as our research that *E. coli* contamination of raw milk samples is a growing challenge.

The outer layer of *E. coli* features many lipopolysaccharides distinguished by the O-antigen. The enucleating of the O-antigen from the wall of bacterial cell attenuate bacterial pathogenicity in suggesting the O-antigen, which plays a key role in host-pathogen interactions [24]. Correspondingly, O-antigens caused antigenic diversity among bacteria and besieged to be the biomarkers for the identification of *E. coli* since the 1940s [25]. Based on the serological analysis targeting the O-antigen of the 10 *E. coli* isolates sampled from raw milk sources in our study, five isolates were serotyped as O111, O27, O114, and O125, and five strains were untypeable by agglutination. The identified isolates were differentiated according to a previous study [26] as enterotoxigenic *E. coli* (ETEC) (O27), enterohemorrhagic *E. coli* (O111), and enteropathogenic *E. coli* (EPEC) (O114 and O125). Our results are related to the results obtained by the previous studies [27,28]. Several authors have recorded a wide range of *E. coli* serogroups in cattle [26,29,30]. Pathogencity of EPEC isolates obtained from milk samples represented a potential risk for public health due to their linkage with infant diarrhea [31-33]. As stated earlier, in this study, five *E. coli* isolates were untypeable in the agglutination test. This highlights one of the challenges of serological methods, as discussed by Delannoy *et al*. [34] who found that the serological agglutination of the O-antigens is difficult, time-consuming, and expensive. Besides, many samples remain untypeable by agglutination; consequently, molecular methods are better alternatives for O-typing [35].

The DNA sequencing of 16S rRNA can aid in the identification of *E. coli* [36], especially in the cases of phenotypic misidentification as occurred with one isolate from the milk samples in our study. The serological evaluation indicated that one isolate was an untypeable *E. coli* strain but was later confirmed as an isolate of *E. hormaechei* (accession no. MN066412). Garbaj *et al*. [37] found that out of 27 *E. coli* strains identified using conventional methods, only 11 strains were confirmed to be *E. coli* using 16S rRNA sequencing. This result suggests the importance of using molecular methods to validate the isolate information obtained from conventional serological techniques.

The additional appraisal of the 16S rRNA sequences of *E. coli* strains earned from milk samples by the neighbor-joining tree found that there was a high degree of similarity between our isolates (similarity of 95.5-100%), as all of these species originated from the *Enterobacteriaceae* family [38]. The sequencing of 16S rRNA further assisted in identifying the five untypeable strains by grouping these strains with GenBank-based reference strains. This analysis revealed that *E. coli* (MN064835) was related to two reference *E. coli* strains (MN006357 and KY711198). Both strains are uropathogenic *E. coli* (UPEC) strains [39]. Moreover, *E. coli* isolates MN066319, MN064861, and MN06483 were found to be related to a reference strain (*E. coli* AP019675), which is an extended-spectrum b-lactamase *E. coli* (ESBL) strain [40]. Most of the reference strains were isolated from patients and environmental samples (e.g., water and soil) [39,40]. Thus, finding similar *E. coli* isolates in raw milk are unsurprising. Nevertheless, this observation highlights the importance of performing a microbiological analysis of milk to verify that there is no public health risk to consume the milk. This is particularly important to curb the outbreaks of food-borne illnesses, which were formerly linked to the consumption of milk and dairy products that had been contaminated with pathogenic bacteria or their toxins [41].

STEC (Stx) strains were identified from cattle farms more than 3 decades ago. Studies implemented in several regions have shown that 10–80% of cattle may harbor STEC [29,42,43]. In this study, the *stx2* gene was found in only one isolate (untypeable *E. coli*), whereas the *stx1* gene was not harbored by any of the 10 isolates. Our result matches the previous studies [28,44] but differs from the results obtained by other studies [45,46]. Almost the equal distribution of *stx1* and *stx2* was noted by Zschock *et al*. [47] in the fecal samples of *E. coli* obtained from cows. This may be attributed to the repeated turnover of serotypes of *E. coli*, with many being isolated only sporadically from a herd [48]. Furthermore, Jenkins *et al*. [49] confirmed that there is a dissimilarity in the percentage of *stx* genes detected, according to the distribution of STEC.

As it pertains to pathogenesis, *E. coli* utilizes various virulence factors to colonize and infect host cells. One factor is intimin (encoded by the *eaeA* gene), which plays an important role in the pathogenesis of attaching-and-effacing lesion, which facilitates the colonization of host tissues [50]. In our study, the *eaeA* gene was detected in four *E. coli* isolates (40%). Two isolates were EPEC of serotype O125 and O114, and the other two isolates were untypeable *E. coli*. Many authors have previously revealed that EPEC strains harbor the *eaeA* gene [51,52]. The *eaeA* gene was detected in 4% of *E. coli* isolates in a previous study [46] and 25% of isolates in a study by Hussien *et al*. [53].

A second key virulence factor used by *E. coli* to promote pathogenesis is the liberation of hemolysin, a pore-forming cytolysin (encoded by the *hlyCABD* gene). In our study, we illustrated that only one isolate belonging to EPEC harbored the *hly* gene. EPEC strains, UPEC strains, ETEC strains, and STEC strains carried the *hly* gene, which is encoded on a plasmid reported by Burgos [54].

After evaluating the existence of genes encoding key virulence factors, we next assessed the antibiotic resistance profile of the 10 *E. coli* isolates obtained from the raw milk samples. The propagation of antibacterial resistance is the leading cause of many health problems worldwide. In this study, we found that a high percentage (100%) of *E. coli* isolates were resistant to penicillin, oxytetracycline, doxycycline, rifampicin, trimethoprim/sulfamethoxazole, and tetracycline. Multidrug resistance (resistance to more than 3 classes of antibiotics) was also observed in several of our *E. coli* isolates. The isolation of multidrug-resistant *E. coli* isolates is not surprising as this phenotype has been reported for *E. coli* in other studies [55,56]. In addition, two studies have found that *E. coli* isolates obtained from milk samples were resistant to antibiotics, mainly to b-lactam antibiotics [57,58].

Bacterial resistance to antibiotics may occur by an impulsive mutation in the target gene(s) due to using antibiotics in therapy or enhancing growth in animals. These antibiotic-resistant bacteria can be spread in the environment when infected animals defecate, thus contaminating soil and agricultural products. If the raw products are consumed, then the resistant bacteria may consequently colonize and infect humans [59]. Among the bacterial isolates obtained from the raw milk samples in this study, we found that genes conferring resistance to tetracycline (*tetA(A)* and *tetB*) were the most prevalent. This finding is concerning because tetracycline is one of the most important antibiotics used in Egypt [60,61].

In addition to resistance to tetracycline, the presence of ESBL’s confers resistance to many antibiotics, such as aztreonam, related oxyimino-b-lactams, cephalosporins, and penicillin. There is a wide variety of ESBLs, including plasmid-mediated resistant to ampicillin, oxacillin, and cefotaxime [62]. The mechanism of drug efflux pumps and the existence of the outer membrane further obstruct the entry of antibiotics in Gram-negative bacterium such as *E. coli*. Focusing on ESBLs, in this study, the *blaTEM* gene was detected in all *E. coli* isolates. The prevalence of *blaTEM* found in our *E. coli* isolates matches results obtained by the previous studies [63,64], who found that most of their *E. coli* isolate harbored *blaTEM*.

Our study demonstrated a relationship between the resistance to drugs and the presence of virulence factors. The attainment of antibiotic resistance and virulence factors in *E. coli* can arise through gene transfer in the environment or the human and animal guts. The genes can be found either on the same plasmid or separately on bacterial chromosomal DNA and plasmids. These genes might be coselected in response to antibiotic pressure, hence aggravating the hazard imposed by *E. coli* on food safety [65,66].

## Conclusion

According to this study’s findings, *E. coli* is considered one of the most common food-borne pathogens that can be transmitted to humans by drinking raw milk. Some strains of *E. coli* harbor some virulent genes *eae*, *hyl stx2* genes, antibiotic-resistant genes *blaTEM*, *tetA*(A), and *tetB*. Furthermore, they showed marked resistance to several antibiotics commonly used in animals and humans in Egypt (MDR). Therefore, much more attention should be paid to hygienic measures during the milking process in dairy farms and the prudent use of antibiotics supported by antibiogram tests before drug administration in dairy farms.

## Authors’ Contributions

WY and HMAM: Designed the study, drafted and critically revised the manuscript, collected samples, and did laboratory works. SH: Revised the manuscript and interpretation of the data. All authors read and approved the final manuscript.
